# Health professionals learning qualitative research in their workplace: a focused ethnography

**DOI:** 10.1186/s12909-020-02191-5

**Published:** 2020-08-17

**Authors:** Luca Ghirotto, Ludovica De Panfilis, Silvia Di Leo

**Affiliations:** Azienda USL - IRCCS di Reggio Emilia, Viale Umberto I°, 50, 42123 Reggio Emilia, Italy

**Keywords:** Continuing learning, Continuing medical education, Healthcare professionals, Professional development, Qualitative Research methods, Research skills, Clinical Cancer center, Cancer Research

## Abstract

**Background:**

The interest for qualitative research methodology has expanded beyond theoretical academic research on medical education, gathering interest from all healthcare professionals. Qualitative research has potentials in exploring the social, emotional, psychological aspects of care and in broadening professionals’ scientific competencies. Nonetheless, qualitative research has still not been embraced within formal and academic curricula for future professionals, preventing newer generations from appreciating the value of its epistemological and methodological aspects and from using it in the development and implementation of clinical research. The purpose of this study was to comprehend the attitudes of health professionals learning and conducting qualitative studies within a practical training program developed in their workplace.

**Methods:**

The present work consisted of a focused ethnography, including 14 professionals during their one-year attendance training on qualitative research methodology. Strategies used for collecting data included participant observations, field notes, semi-structured interviews, and a focus group. All the data were analyzed consistently with ethnographic indications.

**Results:**

Analyses allowed us to evidence the educational, motivational, group-related and organizational factors influencing the attitudes and skill acquisition of healthcare professionals learning and conducting qualitative research within a practical training program developed in their workplace. Prior educational background was perceived as a sort of barrier. Nonetheless, the training boosted a change in attitude both in terms of appreciation of the research approach and trainees’ emotional involvement with research participants. Doing a qualitative study in a multidisciplinary team raised in-group dynamics that hindered bringing the studies to conclusion. Trainees repeatedly lamented the difficulty in managing time to devote to research-related activities and questioned the feasibility of adopting this methodology for conducting research in their workplace.

**Conclusions:**

Continual education training on the methodological aspects and practical implications of qualitative research may foster a renewed attitude towards one’s professional education, while making inter-professional relationship issues emerge. Nonetheless, broadening the perspectives of professionals on their clinical practice by means of learning qualitative methodology may have an evident quality improvement return. Strategies for future qualitative research methodology hands-on training addressed to health professionals in continuing education are proposed.

## Background

In recent years the interest for qualitative research methodology and methods (QRM) has gradually expanded beyond the mere theoretical academic research on medical education, gathering interest from all healthcare professionals (HPs) [1–3] thanks to its potential in exploring the social, emotional, psychological aspects of care [4] and in broadening professionals’ scientific competencies. Nonetheless, QRM has still not been embraced within formal and academic curricula for future HPs [4–7], preventing newer generations from appreciating the value of its epistemological and methodological aspects and from using it in the development and implementation of clinical research. Charmaz highlights the limited coverage of qualitative research in general methods courses, non-existent or insufficient undergraduate courses and a scarcity of in-depth qualitative methods courses in graduate programs [8].

Accordingly, evidence on HPs’ training in QRM is also sparing and limited to the academic environment [9–11], with few works addressing QRM in Continual Medical Education (CME) and its application in HPs’ workplace [12–15]. A study performed by Calderón describes an online teaching program for primary HPs, highlighting specific aspects that should be considered in the design and development of QRM training within health services [12]. Featherstone and colleagues [13] as well as Hepworth and colleagues [14], reporting on their experience with primary care and general practitioners respectively, provide information on which areas of QRM HPs consider most feasible (i.e., coding) or more challenging (i.e., QRM philosophy and theoretical underpinnings) [12, 14]. Authors highlight the career paths, time constraints, and professional background that influence the way HPs approach QRM training [12]. These studies addressed concise QRM training programs and did not explore the experience nor the benefits perceived by HPs in employing QRM within their research activities. As far as we know, no studies exist on the real experience of HPs performing qualitative research.

Herein we report the findings from an ethnographic research on a one-year QRM training to enable HPs to perform qualitative research within their work environment.

## Methods

### Methodological approach

We employed an ethnographic approach previously used by Ng et al. for studying HPs education and practice [16] and recently discussed as a suitable approach in medical education research [17]. In particular, we followed a specific method defined in scientific literature as rapid ethnographic research [18] or focused ethnography [19]. Ethnography represents a validated methodological approach to study personal experiences and behaviors through the direct observation of situations as they enfold in their natural setting [19]. Since ethnographic research is exploratory in nature, in general, research questions are not necessarily specified [20]. Equally, focused ethnography (FE) entails entering the field with a defined research question [17, 18], undertaking fieldwork in a short timeline [19]. Furthermore, compared to traditional ethnographies, FE draws more heavily on interviewing than on participant observation [17]. This method was consistent with medical education ground, which consists of limited and well-defined social episodes or scripted interactions [17]. For our study’s purposes, we formulated the following generative research question: what are the attitudes of HPs towards learning and conducting QRM within a practical training program developed in their workplace?

### The organizational setting

The present work was carried out in a Clinical Cancer Centre (Azienda USL – IRCCS, the public health authority of the Reggio Emilia province) in northern Italy, in the setting of a qualitative research training course, entitled “Carrying out qualitative research: an opportunity for health professionals”. The course was advocated by the Scientific Director to accomplish one of the mission goals of the Centre, i.e. developing and implementing training programs aimed at improving HPs’ research competencies. Motivations supporting the development of such a training within a Clinical Cancer Centre concern the importance QRM for understanding cancer care in its complexity. As noted elsewhere [21], cancer researchers have been increasing their focus on patient preferences and experiences with care. An understanding of how these areas affect oncology practice required HPs working within the Centre to learn research methods suitable to develop a more comprehensive knowledge of the cancer-related phenomena, including methods to describe and explain underlying motivations and potential causes of specific outcomes [21]. Even if the training was endorsed by the Scientific Directorate of the Clinical Cancer Centre and was addressed primarily to the Clinical Cancer Centre professionals, it was opened to all the employees of the main General Hospital and other services of the public local health authority, Azienda USL – IRCCS of Reggio Emilia (including colleagues working at the Department of Health Sciences of the University nearby).

### The training

The QRM training program was designed and conducted by a qualitative research methodologist (LG) and a psycho-oncologist expert in QRM (SDL). Its structure and duration have been planned in line with two strictly interconnected objectives, i.e. providing HPs with notions and skills in QRM and getting them able to carry out qualitative research in all its steps, from the development of the research protocol to its concrete implementation until the publication of the study results.

Instructors chose to teach QRM through the lens of a particular research tradition: Grounded Theory (GT). Some authors [22] recommend GT for hands-on skill-building lessons to impart the fundamentals of qualitative inquiry and methods of inductive theory construction. Moreover, as remarked by Charmaz [8], GT strategies enable trainees to learn to do qualitative analysis, to raise the analytic level of their qualitative research projects, and to increase their methodological skills. Moreover, teachers chose GT given their expertise in this methodology and, consequently, facilitating trainees’ involvement in conducting real qualitative research.

Participation to the training was voluntary, and all interested candidates from any professional background e-mailed the teachers a brief resume, adding, as requested, some information on their motivation in taking part in the program. Selection criteria were having basic comprehension in research methodology and intermediate-level English knowledge. Participants - 15 HPs working at the Clinical Cancer Centre, the Local Health Authority, or the Department of Health Sciences of the University nearby - were identified by the teachers together with the Scientific Director. Their initial motivations to take part in the training included curiosity toward the topic of QRM, willing to complete their previous research-related training and to be involved in the Scientific Directorate research programs.

The training started in October 2015 and had a planned duration of 12 months, so that trainees could accommodate both training’s requirements and their job responsibilities. According to the training’s objectives, teaching was carried out through multiple didactic techniques (i.e., lecture, classwork, working groups, individual work, simulations, and practice), and an e-learning platform was also available. Trainees were committed for 120 training hours (30 h for attending lectures and individual study, 70 h for simulations, teamwork and research practice within small groups; 20 h of individual research-related work). All the time spent within the program has been recognized as part of the working hours. Every participant would have earned 50 CME credits after training’s completion.

Table 1 summarizes the course’s syllabus excerpt outlining contents, performed activities, related teaching methods, and expected outputs. HPs could consult teachers at any time, beyond formalized sessions.

**Table 1:** – “Carrying out qualitative research: an opportunity for health professionals” syllabus (excerpt)

#Phase and *month*	Content	Activity	Didactic method	Expected output
**#1** ***month 1***	Introduction to qualitative research and GT, along with the value of the research question	Teachers explain how to define a GT research question and trainees write a possible one	Lecture	Drafting a research question
**#2** ***month 1***	Is my research question new?	Every trainee performs a literature search	Individual work	Redefinition of the research question
**#2** ***month 1***	The research question in GT	Teachers discuss each research question, and choose the three that are correct and most feasible	Class discussion, guided by teachers	Formation of three research groups and definition of the PI
**#2** ***month 2***	The groups’ research questions	Each group, guided by PI, re-defines the research question	Group discussion without teachers	Reaching an agreement
**#2** ***month 2***	The groups’ research questions	Each group PI explains and discusses the final version of the research question	Class discussion, guided by teachers	Redefinition of the research question
**#2** ***month 2***	Writing a GT research protocol for the EC	Teachers explain how to write a GT research protocol and the local EC requests	Lecture	Definition of the protocol contents
**#2** ***month 2***	The qualitative interview	Every trainee studies material about qualitative interviewing and keeps writing the research protocol	Individual work	Drafting possible themes for the interview
**#2** ***month 3***	The qualitative interview guide	Teachers counsel each group regarding the proposed interviews	Group discussion with teachers	Shared definition of the interview guide
**#2** ***month 3***	Open coding	Teachers explain what GT coding is and invite each group to open code an interview sample	Class work, tutored by teachers	Completion of the interview open coding
**#2** ***month 3***	Focused coding and the inter-coders’ agreement	Teachers invite each group to reach an agreement on the focused coding of the interview sample	Group work, tutored by teachers	Reaching an understanding of focused coding
**#2** ***month 3***	Ethical aspect of qualitative research	Teachers explain how to accomplish research ethics and provide information about the documentation to be presented to the EC	Lecture	Writing informed consent and collecting the documentation requested by the EC
**#2** ***month 4***	Writing the research protocol	Each group finalizes the writing of the protocol	Individual and group work	Submission of the research protocols to the EC
**#3** ***month 7***	The initial sampling	Each group gets access to the field and collects the first interviews	Individual and group work	Having at least 7 interviews conducted and transcribed
**#4** ***month 8***	Open coding	Each group codes the first interviews	Individual and group work, tutored by teachers	Open codes
**#4** ***month 9***	Focused coding	Each group discusses the provisional focused coding with teachers	Group work, tutored by teachers	Definition of focused categories. Each group keeps interviewing and coding
**# 5** ***month 9***	Theoretical sampling and saturation	Teachers define the method for following the theoretical sampling with trainees and explain saturation	Lecture and group work, tutored by teachers	Definition of the final sample characteristics
**# 5** ***month 10***	Final data collection and analysis	Each group performs focused coding on the data collected	Individual and group work, tutored by teachers	Reaching saturation
**#5** ***month 11***	Theoretical coding	Teachers explain theoretical coding and the procedure for working theoretically with the data	Lecture and group work, tutored by teachers	Drafting the theoretical model
**#6** ***month 11***	Findings	Each PI reports the findings	Class discussion, guided by teachers	Having the findings validated by colleagues
**#6** ***month 12***	Writing the report	Each group writes the final research report and documents the steps of the research process for the EC	Individual and group work, tutored by teachers	Having 3 reports written

Once trainees individually developed their research question (phase 1), teachers selected the three most relevant, innovative, and feasible questions and then divided participants into three groups, asking them to conduct a GT study, beginning from the three research questions. The trainees who initially formulated the selected research questions assumed the role of principal investigators (PIs), and the teachers chose their team members according to similarities in research’s interests. The three generative questions were: a) what is going on from patients, clinicians and other key-informants’ point of view when HPs propose surgery to a head and neck cancer patient?, b) what happens when hospital HPs accompany for the first time a patient to death?, c) what is the hospital assistance of migrant cancer patients? To date, the group dealing with the first research question published the study [23], while the other two groups submitted the manuscripts only recently.

### Sample and participants

Participation in the training implied involvement in the FE. The participants in this study comprised 14 trainees (7 nurses, a speech therapist; a laboratory technician; the information specialist of the Medical Library; a dietician; a physiotherapist; an internist and a palliative care physician) and a trainer. Three out of the trainees were PIs of the GT research protocols (the internist, a nurse, the speech therapist). One trainee withdrew from the course after the opening lecture. We show the participants’ demographics and characteristics in Table 3.

### Data collection

The data collected for this FE regarded the trainees as they were attending the course and undertaking the GT study. An external researcher (LDP) participated as the observer to 12 of the 20 training sessions foreseen in training, in order to gain a complete representation of the trainees’ experience.

As to strategies used for collecting data, we employed unobtrusive participant observations consisting of simultaneously combining interviewing of trainees, direct participation and observation, and introspection [24]. Participant observation allowed a prolonged social interaction among the researcher and the informants during which observational field notes were collected. The field notes provided context and insights to support the understanding of what trainees thought about and how they experienced the QRM training. Informal conversations with participants were, also, documented by LDP, who in particular observed relational dynamics, verbal and non-verbal behaviors of trainees. LDP wrote notes about what was going on during the training sessions, the spoken words of teachers and trainees, on her research diary, as much as possible in real time. Informal conversations were jotted down soon after their conclusion. Then, periodically, LDP wrote a more detailed description of the observational sessions (which are specified in Table 2).

**Table 2:** Data collection timeline during the QRM training

#Phase and *month*	Activity	Data Collection Method
**#1** ***month 1***	Teachers explain how to define a GT research question and trainees write a possible one	Observations and field notes
**#2** ***month 1***	Every trainee performs a literature search	Observations and field notes
**#2** ***month 1***	Teachers discuss each research question, and choose the three that are correct and most feasible	Observations and field notes
**#2** ***month 2***	Teachers explain how to write a GT research protocol and the local EC requests	Observations and field notes
**#2** ***month 2***	Every trainee studies material about qualitative interviewing and keeps writing the research protocol	Observations and field notes
**#2** ***month 3***	Teachers explain what GT coding is and invite each group to open code an interview sample	Observations and field notes
**#2** ***month 4***	Each group finalizes the writing of the protocol	Observations and field notes
**#4** ***month 8***	Each group codes the first interviews	Observations and field notes
**#4** ***month 9***	Each group discusses the provisional focused coding with teachers	Observations and field notes
**# 5** ***month 9***	Teachers define the method for theoretical sampling with trainees and explain what saturation is	Observations and field notes
**#5** ***month 11***	Teachers explain what theoretical coding is and to the procedure for work theoretically with the data	Observations and field notes
**#6** ***month 12***	Each PI reports the findings	Observations and field notes
		Focus group
		Interviews

Towards the end of the training, LDP administered semi-structured interviews with the three PIs, aiming to explore their experience in leading the team, expectations, and the evaluation of the training. We chose to interview PIs since we considered them as key-informants in shedding light on the experience of becoming thoroughly involved in all the aspects of performing research, including leading a team, supporting team members’ competencies, and maximizing intra-group collaboration. At the end of the training, the same researcher conducted a focus group, guiding the discussion around the following topics: initial motivation and how it evolved throughout the course, difficulties encountered, and overall evaluation of the experience. We report the timeline for data collection and specific time points for each method in Table 2.

### Data analysis

LDP began the analysis while collecting field notes, as recommended by Roper and Shapira [25] and Hammersley and Atkinson [26]. The field notes were complemented with analytical comments. After their conduction, then, both interviews and the FG were audio-recorded and verbatim transcribed. The researcher managed the field notes similar to the interview and FG data: the dataset was inductively analyzed employing triangulation, with the intent of providing a more in-depth and holistic understanding of the phenomenon [19]. This approach also sustained the comparison of emerging patterns across the different kinds of data (interviews, field notes, and FG). The analysis included a five-step process [27]:

(i.) sorting of collected material (field notes, interviews and FG transcripts were read extensively);
(ii.) descriptively coding of field notes, interviews and FG (LDP descriptively labelled the data, with codes’ names close to the raw data. The researcher reviewed all data line by line and identified words, phrases, and events);
(iii.) questioning of data to find similarities and differences (LDP organized codes and formatted them into tables according to data type. Then, she wrote descriptive accounts of the data from the different sources. Through meetings, authors reached an agreement about the main similarities and differences emerging during this step);
(iv.) grouping of codification labels into behavioral patterns (LDP grouped codes into categories. The emerging behavioral patterns were compared with raw data and descriptive accounts);
(v.) interpreting, through comparison of participants’ meanings and categories (categories were regrouped into the following main factors: educational, motivational, group-related and organizational factors) and defining final conceptualization (authors added meaningful quotations and field notes excerpts to the final report).

### Reflexivity

The external researcher of this study has a master’s degree in Philosophy, an MD in Palliative Medicine, and a PhD in Medical Bioethics. She had previous experience in conducting ethnographic studies within her PhD project. She engaged actively with research participants, trying to balance the inside versus outside continuum in conducting observations, collecting data, and interviewing participants. Her philosophical background influenced data analysis, as she might have been more likely to notice and highlight relational issues emerging from learning QRM. LG and SDL served as consultants and discussants during the data collection and analysis. LG holds a MA in Education and a PhD in Cognitive and Education Sciences, during which he deepened the qualitative methodology. He serves as methodologist and head of Qualitative Research Unit within the setting where this study was conducted. SDL is psychotherapist with expertise in oncology and research methods. She works at the Clinical Cancer Center as the coordinator of the Psycho-oncology Unit.

## Results

LDP carried out the ethnographic observations throughout twelve sessions, over a 12-month period. The observations lasted two hours for each session on average. All three PIs agreed to be interviewed, and each interview had a mean duration of 25 min. The final focus group meeting, which lasted 120 min, was attended by 6 of the 14 trainees (two physicians, three nurses, one laboratory technician), and by one of the two instructors. Table 3 shows the characteristics of the trainees who participated in the study.

**Table 3:** Participants’ characteristics (trainees)

Role	Profession	Gender	Age	Education	Work Place
**Trainee/researcher**	Information specialist of the Hospital Medical Library	F	41–50	Humanities	Clinical Cancer Centre
**Trainee/researcher**	Physiotherapist	M	31–40	Rehabilitation Sciences	Local Health Authority
**Trainee/Principal Investigator**	Professor of Scientific Evidences for Nursing	M	61–70	Nursing	University
**Trainee/researcher**	Dietician	F	31–40	Nutrition Sciences	Local Health Authority
**Trainee/researcher**	Nurse, responsible of Palliative Care Unit Training	F	51–60	Nursing	Clinical Cancer Centre
**Trainee/researcher**	Palliativist	F	31–40	Medicine	Clinical Cancer Centre
**Trainee/researcher**	Nurses’ Manager	F	41–50	Nursing	Clinical Cancer Centre
**Trainee/researcher**	Nurse	F	31–40	Nursing	Local Health Authority
**Trainee/Principal Investigator**	Oncologist	F	31–40	Medicine	Clinical Cancer Centre
**Trainee/researcher**	Laboratory Technician	F	31–40	Neurophysiopathology Techniques	General Hospital
**Trainee/Principal Investigator**	Speech Therapist	F	31–40	Speech Therapy and Rehabilitation Sciences	University
**Trainee/researcher**	Nurses Manager	F	51–60	Nursing	Local Health Authority
**Trainee/researcher**	Physiotherapist	F	41–50	Rehabilitation Sciences	University
**Trainee/researcher**	Physiotherapist	F	31–40	Rehabilitation Sciences	General Hospital

Educational, motivational, group-related and organizational factors were identified as influencing the attitudes and skill acquisition of HPs learning and conducting QRM within a practical training program developed in their workplace.

### The role of the educational background

Prior educational background was perceived as a sort of barrier. In most cases, participants voiced that their background in evidence-based medicine had indirectly prevented their interest and involvement in QRM before, and that they had embarked in this experience with few expectancies. During the first meeting, teachers collected trainees’ initial expectations: two participants (the laboratory technician and the dietician) have never heard of QRM, the nurses’ manager and a nurse who had previously conducted qualitative studies, and the others declared to have some hint. Most of the trainees stated that they had a deep curiosity and that they wanted to understand if the qualitative results of their research would be applicable in the workplace. However, none of the trainees was sure that qualitative research could be a rigorous research method. Introductory lectures were informally commented to be exciting but vague. GT methodology, in particular, was stated as having a “philosophical flavor” perceived distant from what some of the participants were used to.

Participants gathered for the first lecture seem to pay attention to the content. There is silence. Most of them are nodding when the teachers explain what qualitative research is and why to carry out qualitative studies in health field. When Grounded Theory is introduced, two health professionals raise their hands and question “what does it mean we are supposed to theorize?” (*Excerpt from field notes, month 1*)

A nurse states: “I come from a purely quantitative background, so it will be difficult for me to switch to a different mind-set! The contents we discuss are difficult to understand!”. (*Excerpt from field notes, month 2*)

During the second meeting, where each participant had been requested to share with the whole group a generative research question previously conceived, and received feedback by teachers, some trainees proposed questions for a GT method, while others proposed vague or not appropriate ones. The teachers did not find fitting a GT, the research questions about technical procedures (for example, “how is the neurosurgery implemented when a neurophysiological monitoring is available?” or “is the infusion of chemotherapy that causes serious side effects managed properly by nurses?”), with hypothesis-driven pre-assumptions (“how do family dynamics affect the choice to undertake a profession in the health sector?” or “why do healthcare professionals read little?”), or, finally, too vague and far-reaching (“how does the awareness in health professionals regarding the idea of their ‘limit’, illness and death change?”). Even during the fourth meeting, some trainees were still confused about one of the chosen research topics (i.e. the second generative research question we mentioned above).

During the following meetings, it was observed that trainees repeatedly asked the teachers for clarifications on concepts and vocabulary used. Some participants, especially those with a strong quantitative background, continued to express their doubts about the epistemological legitimacy of the label “scientific” for QRM. These persisting questioning pushed teachers to discuss the methodological and epistemological QRM underpinnings till trainees submitted the research protocols to the Ethical Committee. Discussions were often about the meaning of conducting a naturalistic inquiry in health, the interpretative turn, the role of human and social sciences in comprehending health-related phenomena, and the constructivist research paradigm. Moreover, observations have been made about participants needing to understand what they had to do during the phases of drafting their interview guides for the protocol and the phases of coding. The tasks the teachers planned to accomplish have been perceived too broad.

[During a group meeting on interviews coding] the teacher asks the participants to conceptually code the data, word by word or incident by incident if convenient. The nurse asks: “how many labels you want from this interview?”. The PI doubts the group can do this. The teacher says he is available for comment on their provisional coding. (*Excerpt from field notes, month 8*)

Trainees preferred support from time to time in focusing their work on a specific aim.

At the end of the training, most of the participants gained a sense of trust toward the scientificity and rigor of non-quantitative evidence construction, still revealing not to be autonomous in performing activities like defining the research question, the interview guide and the coding. During the final FG meeting, the teacher emphasized this, highlighting positively the effort some of the trainees put into the training activities but also commenting that, in her view, especially people with long-lasting experience in a clinical and quantitative research would need an interdisciplinary team for doing qualitative research. Contextually, the trainees complained about having “wasted time” in the activities related to research question definition, preferring to have had a “prepackaged” research question. This preference emerged mostly from clinicians.

Initial curiosity seemed to turn into pleasure, as stated by one participant during the final FG meeting.

“I have to admit it was a pleasant surprise, even though it is an undefined topic.” (Nurse)

Another trainee evidenced

“The pleasure to look back and reflect over things and observe them in depth!” (Physician).

### Motivational aspects and difficulties in learning and conducting QRM

Motivations and personal investment in the training were observed, since the first meeting, when trainees seemed to have understood that the training would have required a personal commitment. A nurse voiced:

“I know that qualitative research is a demanding task” (Nurse).

Generally, it could be observed that some steps (constructing the research question, performing interviews, and coding) were perceived particularly challenging. One of the PIs reported:

“Constructing the question that drove us to do the study and the search for categories were the elements that seemed most critical to me.” (Nurse).

Difficulties in QRM skill acquisition emerged mainly from interviewing. The members of two groups struggled both to cope with their feelings elicited by the research topics (generating questions a. and b.) and to bracket their sensitivity and professional background. The third group whose research question was ‘what the hospital assistance of migrant cancer patients is’, decided not to interview patients. They feared to live an emotional burden facing “frail” patients. Besides, they were strongly motivated in being involved in the research as they perceived it relevant for local policies.

The majority of the trainees highlighted the emotional difficulties in being so close to research participants during the interviews. From the analysis of the field notes, the recurrent words and phrases used to describe the personal emotions related to interviewing were: “feeling in difficulty”, “I feel uneasy”, “after the interview I felt emotionally upset”, “agitated”, “embarrassed”, “I feel afraid”, “I was anxious all the time”. A nurse stated:

“It was a completely new and unknown experience for me. So, it was a dive into the dark, let’s say... and like all new experiences, they scare you because you do not know how to manage them” (Nurse).

As the training progressed, trainees began to report on their perceived professional and personal growth coming from the process of enquiring on real experiences lived by the study participants; this widely emerged during both formal and informal conversations.

The trainees have highlighted, especially at the end of the course, that the knowledge of a research method not based exclusively on the production of quantifiable evidence has fueled an unusual approach to the relationship. In essence, it was noted by nurses and physicians that deepening experiences of patients and family members, as qualitative interviewing demanded, was usually impracticable in clinical practice.

“It gave me a lot, taught me the right way to relate to the person ... because ... in the end you see their point of view, which in the hospital is always left a little aside. Doing qualitative research has fueled a different type of relationship with the subjects” (Physician).

“This can really change you... it can change your approach with people, with patients ... like in the construction of the interviews above all, that is, I initially asked questions to them too directly, with words a little ... that is ... too direct, too even a bit specialized perhaps“ (Nurse).

Despite the worries, the trainees felt involved in interviewing with pleasure and a sense of fulfilment. But this motivational energy weakened during the subsequent stages of the training. A significant change in personal commitment has been observed from the phase that a trainee has called “desk research” (phase #5). Motivation cooled down, especially after the data collection phase, during which trainees repeatedly complained about the difficulty in managing time to devote to research-related activities because of their busy work schedules and shifts. Also, the instructor participating in the FG confirmed that trainees

“seemed to have prioritized practical activities over … data analysis and reporting, which are part of the research … as important as the other tasks”.

At the beginning of the training, participants were always on time and attended regularly. This behavior changed throughout the training and, during the final sessions, some participants were often absent, or they did not do the assigned homework. During the final focus group, all trainees commented on their experience with QRM as positive, and that the course had rewarded their initial interest in learning a research approach.

“I had no expectations; I approached qualitative research as a *tabula rasa* [clean slate], and all the reasons I had to participate have been met beyond my expectations” (Physiotherapist).

As to the instructor, she commented that she felt comfortable working with the trainees and evaluated the experience of teaching QRM to colleagues in the medical field as positive.

### Group-related factors: teamworking

As the instructor remarked during the final FG, the training was based on participants’ interdisciplinary teamwork as means to perform a GT qualitative study. Although the training emerged as an opportunity for enhancing participants’ personal and professional growth through the interaction between professionals with different background and roles, it also activated specific in-group dynamics.

During the first session, teachers shared with the participants a sort of code of conduct requiring the annulment of professional hierarchies for successfully carrying out a joint project. Nevertheless, at the beginning of the training, we could repeatedly observe that the dynamic shaping the relationships between participating HPs was based more on the different roles they had within their workplaces rather than on the communion of intent. Participants often mentioned that:

“Working with physicians, telling them what to do … it is not the way I am used to. I do not know if I will be able to coordinate the team” (Speech therapist, PI).

Moreover:

“I play a different role every day; it’s kind of … strange to me. When I clearly understood that qualitative research is not done without working in a team, I concentrated on this aspect and learned to appreciate the effort to achieve a shared result, to harmonize ideas, to reach a result together” (Physician, PI).

The PIs were invested in an exclusive responsibility; not all of them felt to be prepared to.

“[As the PI of my research project] it was not easy to manage a group of people with managerial roles; however, trying to achieve a sort of working balance between professional roles and hierarchies was very stimulating. I put myself to the challenge” (Speech therapist).

“At the beginning, I did not want this task, I thought it was too demanding (...) but then the atmosphere that was created, what I learned in managing different thoughts and methods, paid me back for the effort” (Nurse)

“If I think of words that identify teamwork, I can use ‘harmony, positive approach, desire to do, good harmony, energy’” (Physician)

Teamwork was also described as a viaticum for dialogue and collaboration between different professionals. As trainees have often underlined, doing research together meant changing habits in communication among professionals.

“Until now, I had never worked so closely with a doctor! I think it is a way to understand each other and lay the foundations for positive future collaboration” (Nurse).

While the collaboration among team members was fundamental to overcome issues related to protocol writing and interview-related worries, as to time for data analysis and reporting, teamwork was also the scenery of arguments among members.

“Fortunately, I had people at my side who helped me a lot, and then slowly, the anxiety and fear have faded and instead has given way to the curiosity of ... precisely being part of something I had never been part of” (Nurse).

The members are very serious at the beginning of the meeting. The teacher welcomes all and asks how the analysis went so far. Participants look at each other. The PI starts saying they need an external point of view because they did not reach an agreement about categories and the theoretical model. The teacher reminds us that all the statements from the data have to be justified by the data themselves. A member says that they stated something that there is not in the interviews. Another one explains that she does not agree. The PI looks at the teacher like saying: you see? (*Excerpt from field notes, month #11*)

In the group led by the nurse, it was possible to notice how teamwork has never been fully realized: after having completed the first data analysis, personalism emerged during the most conceptual analyzes of their GT. The members discussed, in the presence and via email, the possible interpretations of the data and conceptual categories. These discussions became more heated when the group was asked to define a theoretical model, that is, the relationship between the conceptual categories. Each of the members had a different interpretation and wanted it to be accepted.

In the group led by a doctor, on the other hand, it seemed that teamwork has been more peaceful. During the group meetings with the trainers, the PI reported several times that she was satisfied with the teamwork. From the field notes, her leadership emerged as feeble.

In the group led by the speech therapist, that completed the research and first published the article, the members were able to differentiate competencies and tasks and, most importantly, to put aside their personal research interests in favor of that one of the groups. The librarian commented:

“Even if you do not care about the topic, you do not ignore it for reaching the goal. I have to abstract myself from my research interests. Maybe some people are only interested in one aspect, and they know a detail. Sometimes it is better not to know anything. I do not know anything: without prejudice, maybe I am the expert on anything”.

### Organizational factors: the feasibility of Qualitative Research and its relation to the work setting

Beyond educational, motivational and group-related factors which affected attitudes towards conducting QRM and competencies’ acquisition, organizational factors were identified as influencing skills’ deployment. Trainees often questioned the feasibility of implementing QRM in their workplace. Although this issue often emerged during the various phases of the training, observations performed during the last sessions led participants’ beliefs to come to light explicitly. Opinions expressed by trainees toward this topic differed by professional role. Nurses with no managerial responsibilities and non-medical professionals were doubtful concerning the applicability of qualitative research in their workplace, in consideration of difficulties they perceived in putting their acquired QRM competencies into their future practice.

In particular, feeling to be the only one in the workplace to know and appreciate qualitative research affected the perception of QRM being feasible in the future.

“It is difficult to move forward if your colleagues do not understand the benefit of QRM, especially since this type of research requires a considerable amount of energy and dedication. It would be a pity not to be able to use it more” (Nurse in a day hospital).

“Since I work in a lab, [QRM] will certainly not help me because it is a technical field, and I regret that. However, that does not mean it has not helped to open my mind and make me want to try. But the fact is that I am quite sure I am not going to use it anymore.” (Laboratory Technician).

On the contrary, physicians and nurse managers were more optimistic about the application of QRM in their area. This was clear since the first meeting when a physician stated

“I started the training because I had noticed the curiosity of colleagues with a similar research methodology and the need to understand in detail some sensitive issues. Since I work in oncology, I realized that there are several issues that quantitative methods cannot explore”.

During the FG, she confirmed her initial idea:

“I have to say that many of my current research projects are the result of this training” (Physician).

Another physician recognized the similarity between QRM and previous training in narrative medicine and often reported her experience during the training. During the final interview, she stated that

“(QRM) extends my previous training in narrative medicine. I do not think I will have difficulty using it again” (Physician).

For all the participants, what alarmed the most about implementing future qualitative studies was the workload QRM needed. Nonetheless, it was observed that trainees who could rely on the help of the teachers and colleagues expressed the willing to plan other research.

While at the beginning of the training participants did not know how much QRM could help them in their work, during the FG, they made suggestions on the implementation of strategies to raise colleagues’ awareness about the usefulness of the QRM (at the institutional level and within their workplaces).

The perception of QRM feasibility in the workplace also varied according to what was considered a relevant topic by trainees. Informal conversations informed that some of the members of the group dealing with the novices experiencing for the first time the death of a patient took the training as a mere research exercise as the topic would have not substantial implications for the clinical practice. The dietician and the physiotherapist, in particular, were those who doubted the utility of addressing such a research topic. PIs of the other two groups, contrariwise, perceived the topic had value for practitioners. During an informal conversation, the nurse manager said:

“I will use the research findings to modify the care pathway and inform the practices in use”.

## Discussion

The purpose of this study was to comprehend the attitudes of HPs learning and conducting QRM within a practical training program developed in their workplace. Following an ethnographic approach allowed us to illuminate the social actions and interactions that occur within a specific context and, in particular, to emphasize the factors influencing HPs attitudes and skill acquisition. As Kuper and colleagues pointed out [28], aspects of medical education should be investigated as the products of interactions between two or more individuals or groups. This was the case of learning and conducting QRM. This could be seen as broadening the methodological horizons in medical education [17, 28].

Based on our observations and analyses, we found that one of the significant limitations to spreading of QRM is the researcher’s methodological mind-set. Most often HPs were educated to an exclusively evidence-based approach which made them skeptical about the rigorous methodology and usefulness in the medical field, in agreement with findings from previous works [11, 29, 30]. To limit the risk that previous educational background would prevent acquiring skills for QRM, academic curricula should address qualitative methodology, at least by informing about the benefit of using qualitative evidence in clinical reasoning [31].

This educational bias was, however, overcome by educating participants on the purpose of the qualitative approach and the validity and academic rigor of qualitative methodology, in agreement with other experiences in literature [4, 32]. The hands-on experience in carrying out their qualitative research projects in a real setting further corroborated their appreciation for the approach and provided them with a new skill set, including qualitative interviewing and inductive data analysis. It appeared that learning QRM by doing it, and especially by performing a GT [8], within a tutored setting, is an essential and promising pedagogy. Besides, qualitative health research always requires practical activities rather than mere theoretical and abstract discourses [33].

Once participants embraced the new mind-set and mastered their new skills, they recognized the positive effect QRM training had also had on their relationships with colleagues that covered different professional roles. This also led to experience the potential of the team effort in pursuing projects, also beyond the scope of QRM. It is worth a mention the responsibility PIs’ are called to take for managing intra-group dynamics, especially in a context of QRM interprofessional collaboration.

Moreover, learning-by-doing how to design and conduct QRM can be thought as a means for humanizing care provided by HPs. Todres et al. [34] describe the mutual relationship between a person-centred value framework for health care and the implications of the findings of QRM as a systematic whole for humanizing caring practices [34]. They stress that a humanizing emphasis on care requires a particular kind of ‘knowledge for care’ and needs studies with specific epistemological and methodological characteristics. Such characteristics are intrinsic to QRM and require, as our trainees realized, personal involvement, openness to contexts, and sensitive relationship to research participants, especially within cancer care [21]. While Todres and colleagues [34] portray a reciprocal relationship between this framework for care and QRM, we can move forward this concept, proposing a complementary relationship between care humanization and learning-by-doing QRM, about which we hope further research will be carried out in oncological settings.

Finally, the study documented another obstacle in the time constraints of HPs and the future feasibility of QRM training. Given that participants already had busy working schedules, the project required they additionally resort to, or develop, time-management skills, in order to be able to complete the study. This aspect of time-management skills was also described by Calderón [12] in a commentary summarizing an eight-year qualitative online training experience addressed to GPs, and by Featherstone et al. [13] in their ten-year follow up on qualitative research training in primary care. PIs should have the capacity of helping team members in managing time and tasks. Nonetheless, the time qualitative research requires is an element to be taken into consideration in the evaluation of the behaviors of HPs doing research in their workplace. It is quite impossible to foresee how much time a qualitative study needs to be carried out. Facilitators to motivate HPs to attend QRM courses may include defining in advance a formal learning agreement among trainees and their clinical managers in terms of mandate and research topic to investigate. HPs need to have their research-related time recognized as part of their profession. CME credits should be flexibly accredited by managers and at least two colleagues from the same unit or ward should attend the training to avoid sense of isolation.

One last noteworthy consideration is on the transfer of QRM skills and applicability in HPs’ departments or units. In our study, the possibility of achieving this appeared to depend on the perceived work-related relevance of the research topic and the presence of other colleagues sharing an appreciation for QRM. As described by Hunt et al. [35], projecting oneself into performing qualitative research in the future has to do with one’s expectation, or not, to be alone and isolated in playing learned skills. Contextually, from our analysis the importance of connecting research topics to professional practice emerged. HPs’ clinical and work activities inform research interests, so it is inevitable that HPs’ expectations about QRM involve what research can do for helping them in their workplace [12].

Findings from our ethnographic study supported the funding by the Clinical Cancer Center Scientific Directorate of two further editions of the training, involving 34 HPs. The results of the qualitative researches carried out during these training programs were reported within local, national and international events. Trainees had the chance to present their work to them colleagues and managers during a seminar. Their studies were commented by an invited lecture from abroad. This allowed spreading awareness around QRM and the added value of applying this type of research in healthcare settings.

As to research relaunch about HPs learning QRM within the workplace, it would be interesting to isolate sensitive outcomes to evaluate over time. This course was an opportunity for trainees to reflect on the relationship with teammates and the participants of their GT studies (colleagues, patients and family members). Learning how to conduct QRM studies may also mean learning how to deal with feelings, overcome hierarchy issues, work together for a common goal, improve relationships in the workplace. Besides, acquiring QRM competencies may positively affect the personal skills in communicating with patients and users. Further research to evaluate the impact of QRM training not only on participants’ learning but, more importantly, on workplace climate and care quality is suitable.

### Strategies for future qualitative research methodology hands-on training addressed to health professionals in continuing education

In the last decades, an interesting debate has aroused concerning the problem of teaching QRM, or ‘how to teach the tools of an uncertain trade for use on an unknown job’, as synthesized by Mason [36]. This FE adds insights on how to design a future learning-by-doing style QRM training to HPs. Following our main results, we may propose points worth considering.

In general, QRM trainers should provide ways for others to learn doing research by researching. Teachers should also avoid abstract descriptions of methods before the novices understanding of what research is and is about [37]. In this regard, planning a syllabus is recommendable: it would avoid improvisation and allows teachers to make a learning agreement with managers and future participants.

Before the beginning, we may suggest the following strategies:

- Hands-on training requires CME providers to be flexible in allowing varied didactic techniques and a flexible schedule. We suggest the trainers share the syllabus with both CME Scientific Committee and future participants’ managers. For the subsequent editions of the QRM training, we planned to discuss in advance HPs candidates’ participation with managers.
- As to organizational factors, time constraints should be taken into account. Managers, who have agreed on the participation of their colleagues, are aware that doing research is a time-consuming activity. Acknowledging proper CME credits or lightening ordinary workload are facilitating factors. In any case, QRM activities have to be reckoned as working hours.
- Trainers should contact managers and ask them to encourage HPs participating QRM training programs, mainly if these include teamwork education, in order to nurture a positive culture of learning and teamwork within the workplace, in accordance to what suggested elsewhere [38].
- It has been reported [4, 11] that cognitive dissonance is an ingredient of QRM learning and practice to be managed by the trainers and, hopefully, by peers. As several authors pointed out [39–41], experienced mentorship plays a pivotal role in becoming confident on how to conduct qualitative research. The training staff should include methodologists as teachers and trainers and experienced tutees.
- As to group-related factors, we noted that being a PI is demanding. PI has to manage intra-group dynamics, and they should be prepared or, at least, accompanied by trainers. According to our findings, the characteristics of teams that have led to successful participation in teamwork (and positive outcomes for team performance) [38] include: the ability of the PI to differentiate the competencies within the group; the capacity of the members to put aside their personal research interests (and personalism) in favor of that one in common. If the QRM training involves conducting real qualitative studies, deciding who will be a PI is unavoidable. A preliminary workshop about what being PI in a research team means may be advisable. This strategy may limit likely hierarchy-related issues.

During the training, we may highlight the following strategies:

- as to educational factors, we already pled for integrating QRM within academic curricula as taking qualitative evidence is becoming more and more beneficial for clinical reasoning [31]. For future methodological training within the workplace, it is crucial to teach ontological and epistemological essentials since they are prerequisites to comprehend QRM. We suggest discussing ontological and epistemological underpinnings while doing research activities by planning in-between sessions for reflexivity and thoughts-sharing. Experiential learning is advantageous to understand both the philosophical orientation and practical skills necessary to conduct QRM [11]. Moreover, this fashion fits the practice-oriented attitude our participants showed.
- Sessions for freely sharing personal accounts (feelings, comments, opinions) among trainers and trainees should be planned in order to deal with motivational factors. According to the recommendations from a systematic review about HPs’ experience of teamwork education [38], the first meeting should explore participant learning needs and their prior experiences of working in teams before implementing teamwork education programs. Then, from time to time, allowing trainees to voice their perceived difficulties in dedicated debriefing and reflection sessions could be a form of support and a facilitating factor for skills’ acquisition.
- We also suggest keeping the level of trainees’ commitment as high as possible by selecting QRM projects perceived to have a practical value. Also, having process evaluation sessions may periodically re-motivate trainees’ participation.

At the end of the training, we suggest the following strategies:

- PIs and team members should arrange brief meetings within the wards or units to present their research to colleagues and managers;
- Trainers, along with trainees, should organize a CME event as an opportunity to raise awareness about QRM and its value for healthcare.

### Strengths and limitations

Limiting a training assessment to participants’ satisfaction and learning questionnaires, necessary for CMEs, reduces the possibility of taking into account personal and contextual aspects. In this case, we conducted a qualitative assessment through FE to better understand the attitudes of the HPs towards new learning. The results discussed here have allowed us to understand, as trainers, what barriers and facilitators HPs have experienced and how to enhance future methodological training offer. Besides, as recently outlined [17], FE offers a methodological approach tailored to medical education characteristics and should be a part of the qualitative toolkit in medical and health sciences education research.

There are also methodological limits which it is essential to discuss. Only six out of 14 trainees participated in the FG. Tracing the reasons for non-participation was not possible. We can hypothesize that those who participated in the FG were more likely to be satisfied with the training they attended. As one teacher was present during the FG, trainees could be influenced during the discussion. Nonetheless, including a teacher together with trainees enriched our dataset, providing observations from a different point of view. Mostly, collected data were about trainees and teachers appeared rarely in the field notes. Only a researcher could analyze the data. Nevertheless, data collection included triangulation, as the researcher compared data from different sources. The last limitation, and a research relaunch as well, we would like to note the choice of teaching trainees with the means of conducting a GT study. This method was particularly demanding in terms of time, resources and requested preparation and could have impacted the results of this ethnographic study. Nonetheless, it appeared to be the most comprehensive methodology for making the peculiarities of qualitative interpretative research appreciable [8]. Exploring the implication of learning how to conduct GT studies, precisely, rather than another type of qualitative investigations, needs further and comparative analyses.

## Conclusions

Taken together, these findings show an appreciation for QRM among HPs and its added value in exploring the many aspects of the healthcare setting that cannot be studied through quantification. The ethnographic approach provided us with the methodological tools to draw and build upon the wealth of personal experiences collected by HPs throughout their career. Our findings show that QRM training within the workplace elicit a number of factors to take into account. Learning qualitative methodology may foster a renewed attitude towards one’s professional education, while making inter-professional relationship issues emerge. Nonetheless, broadening the perspectives of professionals on their clinical practice by means of learning QRM may have an evident quality improvement return.

The development and application of QRM across clinical practice could benefit from (i) dissemination on QRM value and potential in improving everyday clinical practice through brief seminars, and (ii) coordination with medical management to allow the implementation of QRM training within working hours and within clearly defined healthcare quality improvement strategies.

## Data Availability

We do not have ethics approval to make raw data from this study available for sharing.

## Abbreviations

CME: Continual Medical Education
EBM: Evidence-based Medicine
FE: Focused Ethnography
FG: Focus Group
GT: Grounded Theory
HP: Health Professional
PI: Principal Investigator
QRM: Qualitative Research Methodology & Methods
